# Clinical implementation of electric impedance tomography in the treatment of ARDS: a single centre experience

**DOI:** 10.1007/s10877-018-0164-x

**Published:** 2018-05-29

**Authors:** Serge J. H. Heines, Ulrich Strauch, Marcel C. G. van de Poll, Paul M. H. J. Roekaerts, Dennis C. J. J. Bergmans

**Affiliations:** 1 Department of Intensive Care, Maastricht University Medical Centre+, P. Debyelaan 25, 6229HX Maastricht, The Netherlands; 2 Department of Surgery, Maastricht University Medical Centre+, P. Debyelaan 25, 6229HX Maastricht, The Netherlands; 3 School of Nutrition and Translational Research in Medicine (NUTRIM), Maastricht University, P.O. Box 616, 6200MD Maastricht, The Netherlands; 4 Cardiovascular Research Institute Maastricht (CARIM), Maastricht University, P.O. Box 616, 6200MD Maastricht, The Netherlands

**Keywords:** Electrical impedance tomography, Acute respiratory distress syndrome, Positive end expiratory pressure, Mechanical ventilation

## Abstract

To report on our clinical experience using EIT in individualized PEEP titration in ARDS. Using EIT assessment, we optimized PEEP settings in 39 ARDS patients. The EIT PEEP settings were compared with the physicians’ PEEP settings and the PEEP settings according to the ARDS network. We defined a PEEP difference equal to or greater than 4 cm H_2_O as clinically relevant. Changes in lung compliance and PaO_2_/FiO_2_-ratio were compared in patients with EIT-based PEEP adjustments and in patients with unaltered PEEP. In 28% of the patients, the difference in EIT-based PEEP and physician-PEEP was clinically relevant; in 36%, EIT-based PEEP and physician-PEEP were equal. The EIT-based PEEP disagreed with the PEEP settings according to the ARDS network. Adjusting PEEP based upon EIT led to a rapid increase in lung compliance and PaO_2_/FiO_2_-ratio. However, this increase was also observed in the group where the PEEP difference was less than 4 cm H_2_O. We hypothesize that this can be attributed to the alveolar recruitment during the PEEP trial. EIT based individual PEEP setting appears to be a promising method to optimize PEEP in ARDS patients. The clinical impact, however, remains to be established.

**Electronic supplementary material:**

The online version of this article (10.1007/s10877-018-0164-x) contains supplementary material, which is available to authorized users.

## Introduction

Although mechanical ventilation is a lifesaving therapy, it has some negative side effects such as ventilator associated lung injury (VALI). In the last 15 years, extensive research has been performed on lung protective ventilation. The most important study showing a reduction in mortality in patients with acute respiratory distress syndrome (ARDS) was done by the ARDS network investigators. They highlighted the importance of lung protective ventilation especially by avoiding high tidal volumes and high inspiratory pressures. They proposed a standardized approach to adjust PEEP settings according to changes in oxygen demand [1]. In addition they conceived the ARDS network PEEP/FiO_2_ table as a manual to titrate PEEP in ARDS [2]. This approach however, does not take into account individual patient characteristics that determine alveolar recruitment and recruitability such as body mass index (BMI), lung elastance and pulmonary edema [3, 4]. Consequently, a more individualized approach to determine optimal PEEP and tidal volume setting may be desired to optimize the treatment of patients with ARDS. Therefore, strategies like best compliance, stress index and pressure–volume curves were investigated [5–8]. However, these are all based on global parameters which do not exclude regional overdistension, collapse or atelectrauma, especially in patients with ARDS.

Electrical impedance tomography (EIT) is a non-invasive, non-radioactive, bedside imaging tool, providing functional images with a high temporal resolution. These images are dynamic which allows the operator to follow the response of the lungs to a therapeutic intervention on a breath-by-breath basis [9–15]. Because regional overdistension and alveolar collapse can be visualized using EIT it may be superior to the abovementioned global parameters that have been applied in individual PEEP optimization [16]. Despite its theoretical advantages, EIT has not yet been implemented widely in clinical research and practice. This is probably partly due to the complexity of the analysis and interpretation of the data. EIT is performed on regular basis in our institution as part of routine care to adjust and titrate PEEP in patients with ARDS and acute hypoxic respiratory failure. Aim of the present paper is to report on our initial clinical experience using EIT to individualize PEEP setting in mechanically ventilated ARDS patients, and to determine whether EIT guided PEEP is in agreement with physician set PEEP and the PEEP according the ARDS network PEEP/FiO_2_ table.

## Materials and methods

### Patients

Between January 2015 and August 2016 EIT was performed in 39 adult, mechanically ventilated ARDS patients that were admitted to our 33 bed mixed intensive care unit. Maastricht University Medical Centre+ is a University Hospital which has a total of 715 beds. ARDS was defined according to the Berlin definition [17]. Patient characteristics are presented in Table 1. The selection of the patients was based on the indication for EIT. In our centre we use EIT in patients with difficult mechanical ventilation like patients with ARDS. In our retrospective data we selected 39 patients fulfilling the criteria for ARDS who were sedated and invasively ventilated in a pressure controlled, time-cycled mode (BIPAP or APRV) using an Evita-4 or Evita-XL ventilator (Dräger Medical GmbH, Lübeck, Germany). All patients had no spontaneous breathing activity. Tidal volumes between 6 and 8 ml/kg predicted body weight were pursued. No strict institutional guidelines on PEEP setting are in use and PEEP was set at the discretion of the treating physician based on clinical information like Cdyn, PaO_2_/FiO_2_-ratio, BMI, hemodynamics, etc. according to a lung protective ventilation strategy until the first EIT assessment.

**Table 1:** Patient characteristics

Sex (M/F)	25/14
APACHE II (SD)	29 (8)
Age, years (SD)	65 (15)
BMI (SD)	26 (5)
Onset ARDS, hours (SD)	40 (58)^a^
Tidal volume ml/kg PBW (SD)	7.8 (2.5)
PaO_2_/FiO_2_, mmHg (SD)	147 (61)
*Type of admission*	
Pneumonia (%)	20 (51)
Sepsis (%)	11 (28)
Cardiac arrest (%)	3 (8)
Other (%)	5 (13)
*ARDS*	
Mild ARDS (%)	7 (18)
Moderate ARDS (%)	21 (54)
Severe ARDS (%)	11 (28)

^a^Hours between fulfilling the ARDS criteria and the EIT measurement. Data are expressed as mean ± SD

### Electric impedance tomography

EIT analysis was requested by the treating physician and performed by a trained ventilation practitioner (SJHH). An EIT dedicated belt containing 16 electrodes was placed around the patient’s chest at the fourth or fifth intercostal space at the parasternal line, and connected to an EIT monitor (Pulmovista^®^ 500, Dräger Medical GmbH, Lübeck, Germany) with a frame rate of 20 Hz. Low-pass filter was set at a cut-off frequency of 50/min. To improve contact between the electrodes and the skin, a delayed onset of the measurement of 5–10 min was used after application of the belt. The EIT monitor was connected to the mechanical ventilator to import ventilator data into the EIT monitor.

**Fig. 1:**
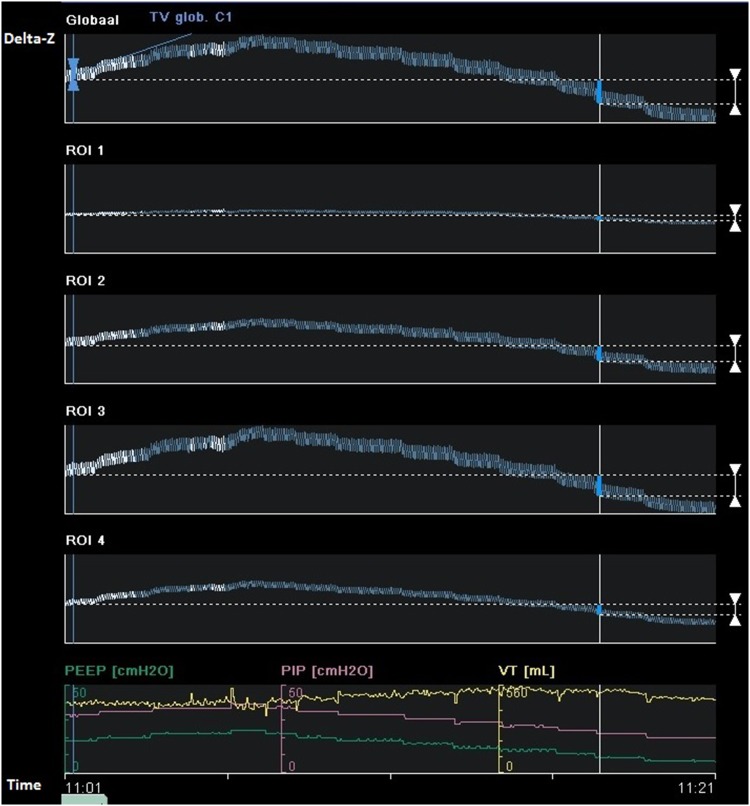
Changes in end-expiratory lung impedance (EELI) values at different decremental PEEP levels reflect the stepwise fall in end-expiratory lung volume (global: overall tidal impedance change; ROI 1 to ROI 4 represent tidal impedance chance from ventral, mid-ventral, mid-dorsal and dorsal region). During the last PEEP step there is a gradual decrease in EELI in ROI 2 reflecting alveolar derecruitment. In ROI 3 and 4 the decrease in EELI starts at earlier PEEP steps (*delta-Z* tidal impedance variations)

**Fig. 2:**
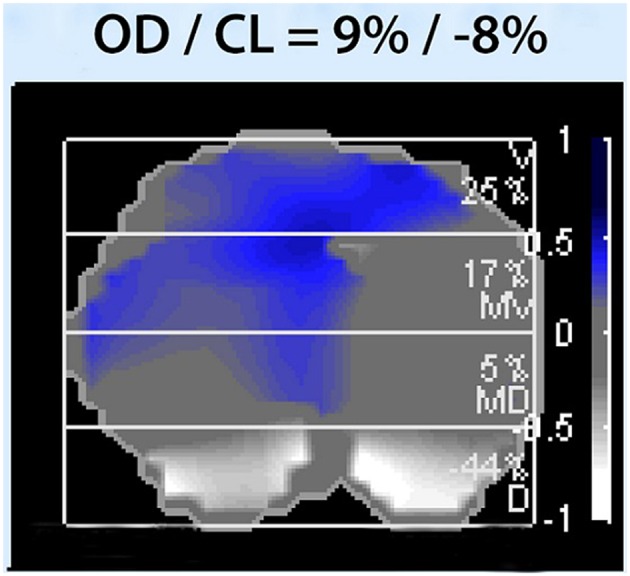
Alveolar overdistension and alveolar collapse (OD/CL)

EIT measurements were performed during an incremental PEEP trial to an average PEEP level of 18–20 cm H_2_O, followed by a decremental PEEP trial, using steps of 2 cm H_2_O. At each PEEP level change in end expiratory lung impedance (EELI) was evaluated. Driving pressure was constant during the PEEP trial. The decremental trial was stopped when loss of EELI was observed, reflecting derecruitment (Fig. 1) Ventilator settings during the PEEP trial are presented. Afterwards, a short recruitment manoeuvre was performed to select the level of PEEP where EELI does not decrease in the dependent lung regions. This is followed by an offline analysis to evaluate the functional EIT images with dedicated software (EITdiag; Dräger Medical). Using this software, tidal recruitment, alveolar collapse, end-inspiratory overdistension and regional compliance could be assessed. Overdistension and CL quantifies the amount of CL and OD in the lung by calculating pixel compliance at every PEEP step. Cumulated OD and CL is determined as the percentage change in compliance for each pixel in relation to its “best compliance” as described by Costa et al. [16]. Optimal PEEP was determined as the best compromise between alveolar overdistension and alveolar collapse by off-line analysis of changes in regional compliance during each PEEP step in the decremental part of the PEEP trial. This means the best regional compliance to maximize recruitment of the dependent lung and minimize overdistension of nondependent lung areas [18, 19] (Fig. 2).

The algorithm calculates for each pixel the compliance deviation of a given section against the highest compliance, which was present during the entire PEEP trial. Blue to dark blue indicates decrease in respiratory system compliance (Crs) caused by overdistension, white indicates decrease in Crs caused by airway collapse. Overall overdistension (OD) and collapse (CL) are given as percentage.

### Clinical application of EIT data and effect evaluation

Ventilator settings were adjusted to meet optimal PEEP derived from the EIT analysis if actual set PEEP differed from this value. The effect of these PEEP adjustments on oxygenation, PaO_2_/FiO_2_-ratio and Cdyn was assessed by comparing values before and after EIT analysis, and subsequent adjustment of ventilator settings.

Arbitrarily, a difference of ≥ 4 cm H_2_O between ARDS network table-based PEEP advise and EIT-based PEEP advise was considered to be clinically relevant. To assess the acute effects of EIT based PEEP adjustment on oxygenation and ventilation parameters, patients were divided in a group where pre-EIT PEEP was < 4 cm H_2_O different from EIT-advised PEEP (group A) and a group where the difference between pre-EIT and EIT-advised PEEP was ≥ 4 cm H_2_O (group B). In addition, we assessed the agreement between EIT-based optimal PEEP level and PEEP level that would have been derived from the ARDS network table [1]. The ARDS network table uses ranges of PEEP, therefore the smallest possible difference in PEEP according the ARDS network table PEEP and EIT-based PEEP advise was used. For example, if the PEEP according the ARDS network table should be in the range of 10–14 cm H_2_O, and EIT PEEP determined to be 16 cm H_2_O, the difference is 2 cm H_2_O.

### Statistics

Data are presented as a number (percentage) for categorical variables and as mean ± standard deviation (SD) or median and interquartile range for continuous variables where appropriate. A paired sample *t* test was used to assess normally distributed data and Mann–Whitney *U* test to assess data that were not distributed normally. A p value < 0.05 (two-tailed) was considered to indicate statistical significance. Two-way ANOVA was used to compare the mean values of Cdyn and the PaO_2_/FiO_2_-ratio before and after EIT measurement. A p-value < 0.05 was considered statistical significant. Mann–Whitney *U* test was used to compare the median values between group A and group B. Individual pair-wise differences between the three PEEP methods were presented by Bland–Altman plots where the size of the dot is an indicator of the incidence of the value. The Bland–Altman plots were made with MATLAB 2017a (Mathworks, Natick, MA). All statistical analyses were performed using SPSS version 23 software (IBM SPSS, Armonk, NY). GraphPad Prism version 5.03 software (GraphPad Software, inc. La Jolla, CA, USA) was used to generate graphs of the individual EELI tracings during the PEEP trial.

### Ethics

EIT is used as part of routine care in our department and indications for EIT were determined based upon clinical grounds. The ethics committee of the Maastricht University Medical Centre+ approved the utilization of the collected data for scientific evaluation and individual informed consent was waived (METC 15-4-186).

## Results

### Feasibility of routine clinical EIT application in ARDS

EIT image acquisition and off-line data analysis was successful in all patients. During the incremental and decremental PEEP-trial no complications such as desaturation, hemodynamic instability or pneumothorax occurred. Ventilator settings during the PEEP trial are presented in Table 2.

**Table 2:** Ventilator settings during the PEEP trial

Delta-P (cm H_2_O)	14.1 (± 4.5)
RR (breaths/min)	24.2 (± 8.8)
Tv high PEEP (ml)	378 (± 135)
Tv optimal PEEP (ml)	553 (± 183)

Data are expressed as mean ± SD (*Delta-P* delta pressure, *RR* respiratory rate, *Tv high PEEP* tidal volume at the highest PEEP level, *Tv optimal PEEP* tidal volume at the optimal PEEP level according EIT)

### Agreement between set PEEP, EIT-based PEEP advise and ARDS network-based PEEP advise

PEEP was set to 11.7 ± 2.6 cm H_2_O in all patients. According to EIT-analysis optimal PEEP would have been 11.3 ± 3.1 cm H_2_O for the entire population which was not significantly different from actual set PEEP (p = 0.305). Actual set PEEP was significant lower in mild versus moderate and mild versus severe ARDS (p = 0.032 and p = 0.042 respectively). EIT advised PEEP was significantly higher in severe versus mild and in severe versus moderate ARDS (p = 0.023 and p = 0.039 respectively) (Table 3).

**Table 3:** Actual set PEEP and “optimal PEEP” based on EIT advise

	Actual set PEEP	EIT based PEEP advise
Total (n = 39)	11.7 (± 2.6)	11.3 (± 3.1)
Mild ARDS (n = 7)	9.7 (± 1.8)	9.4 (± 2.2)^β^
Moderate ARDS (n = 21)	12.1 (± 2.6)*	10.9 (± 2.7)
Severe ARDS (n = 11)	12.4 (± 2.8)^α^	13.3 (± 3.6)^γ^

*p = 0.032 mild versus moderate

^α^p = 0.042 mild versus severe

^β^p = 0.023 mild versus severe

^γ^p = 0.039 moderate versus severe

Actual set PEEP and “optimal PEEP” based on EIT advise. Actual set PEEP was significant lower in mild versus moderate and mild versus severe ARDS (*p = 0.032 and ^α^p = 0.042 respectively). EIT advised PEEP was significantly higher in severe versus mild and in severe versus moderate ARDS (^β^p = 0.023 and ^γ^p = 0.039 respectively). Data are expressed as mean ± SD (Student’s *t* test).

**Fig. 3:**
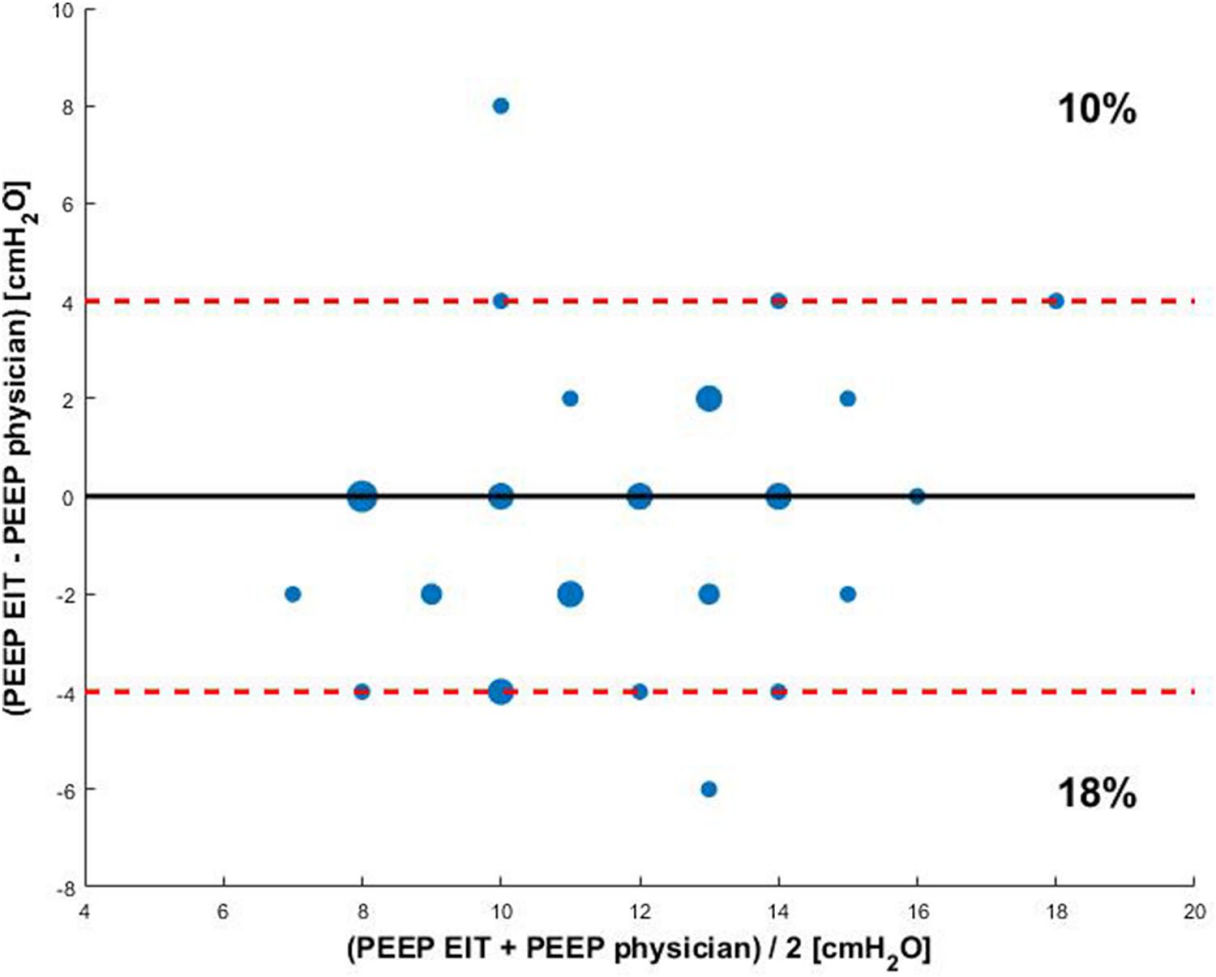
Bland Altman plot of differences in PEEP between EIT guided PEEP and the PEEP set by the physician. The size of the dot is an indicator for the incidence of the value. The red dashed line indicates a PEEP difference of 4 cm H_2_O

Baseline characteristics of ARDS patients between group A and group B are presented in Table 4. In 14 out of 39 (36%) of the cases, EIT-based PEEP advise was equal to the actual PEEP level set by the treating physician. In 11 out of 39 (28%) of the cases the difference between EIT-based PEEP advise and set PEEP level was ≥ 4 cm H_2_O. We observed no systematic higher or lower estimation of desirable PEEP level using one of either approaches. The (dis)agreement between physician set PEEP and EIT advised PEEP is displayed in Fig. 3.

**Table 4:** Baseline characteristics of ARDS patients between group A and group B

	Group A (n = 28)	Group B (n = 11)	p-value
PEEP (cm H_2_O)	12 (4)	12 (6)	0.470
FiO_2_	0.6 (0.4)	0.6 (0.45)	0.355
PaO_2_/FiO_2_-ratio	136 (14)	131 (11)	0.779
Cdyn (ml/cm H_2_O)	44 (30)	34 (32)	0.075

Baseline patient characteristics of ARDS patients where pre-EIT PEEP was < 4 cm H_2_O different from EIT-advised PEEP (group A) and a group where the difference between pre-EIT and EIT-advised PEEP was ≥ 4 cm H_2_O (group B). Data are expressed as median (interquartile range), Mann–Whitney *U* test.

**Fig. 4:**
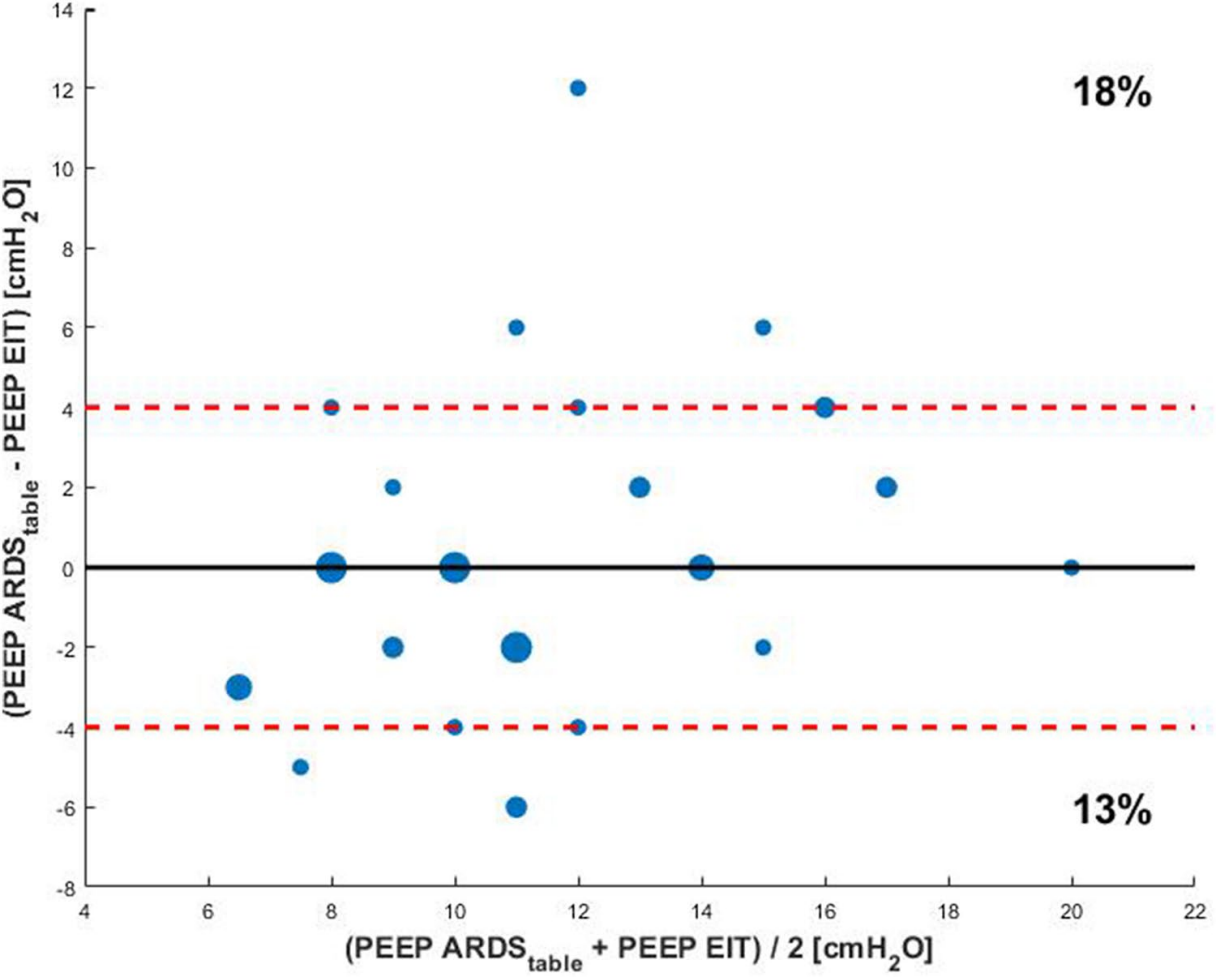
Bland Altman plot of differences in PEEP between the ARDS network table and EIT guided PEEP. The size of the dot is an indicator for the incidence of the value. The red dashed line indicates a PEEP difference of 4 cm H_2_O

EIT-based PEEP advise was in agreement with the PEEP level advised by the ARDS network table only in 12 out of 39 cases (31%) (Fig. 4).

**Fig. 5:**
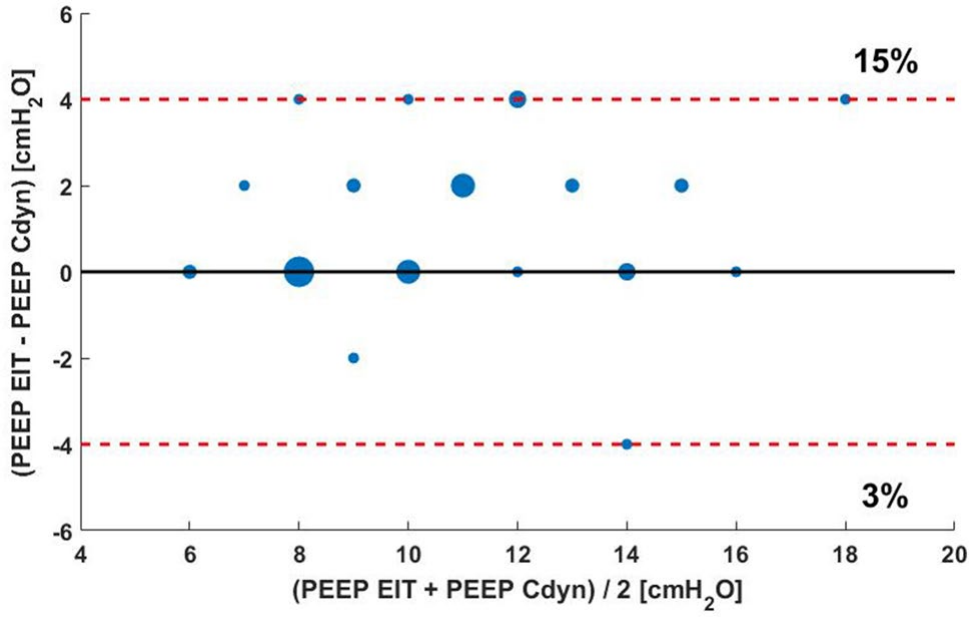
Bland Altman plot of differences in PEEP between EIT guided PEEP and PEEP based on best Cdyn. The size of the dot is an indicator for the incidence of the value. The red dashed line indicates a PEEP difference of 4 cm H_2_O

In 19 out of 39 (49%) of the cases, EIT-based PEEP advise was equal to the PEEP level based on best Cdyn. In 7 out of 39 (18%) of the cases the difference between EIT-based PEEP best Cdyn PEEP level was ≥ 4 cm H_2_O (Fig. 5).

### Acute clinical effects of EIT-guided PEEP adjustment on PaO_2_/FiO_2_-ratio and dynamic respiratory system compliance

**Fig. 6:**
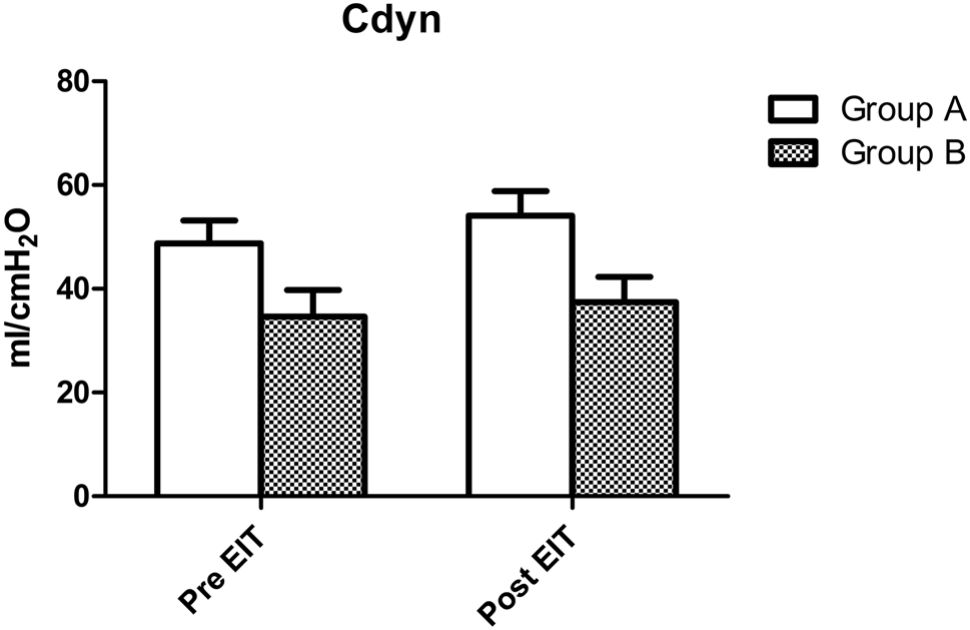
Acute changes in Cdyn in 39 mechanically ventilated ARDS patients after EIT in the group where pre-EIT PEEP was < 4 cm H_2_O (group A) different from EIT-advised PEEP and a group where the difference between pre-EIT and EIT-advised PEEP was ≥ 4 cm H_2_O (group B). There was a significant increase in the Cdyn (p < 0.001), however this increase was not significantly different between both groups (p = 0.151 for interaction, 2-way ANOVA)

**Fig. 7:**
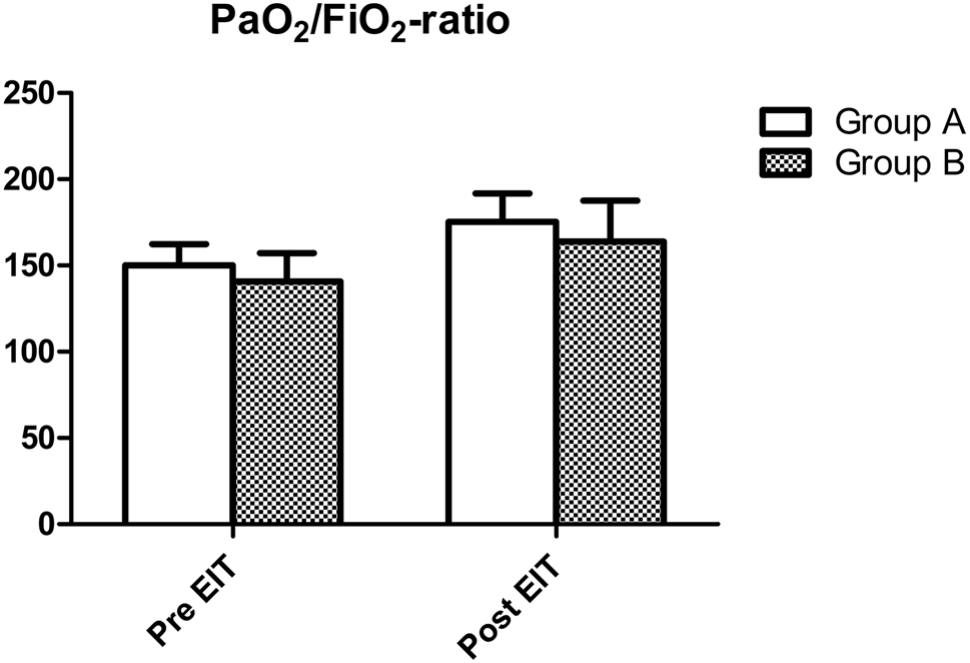
Acute changes in PaO_2_/FiO_2_-ratio in 39 mechanically ventilated ARDS patients after EIT in the group where pre-EIT PEEP was < 4 cm H_2_O (group A) different from EIT-advised PEEP and a group where the difference between pre-EIT and EIT-advised PEEP was ≥ 4 cm H_2_O (group B). There was a significant increase in the PaO_2_/FiO_2_-ratio (p < 0.001), however this increase was not significantly different between both groups (p = 0.894 for interaction, 2-way ANOVA)

As described in the methods section, patients were divided in two groups: a group where pre-EIT PEEP was < 4 cm H_2_O different from EIT-advised PEEP (group A) and a group where the difference between pre-EIT and EIT-advised PEEP was ≥ 4 cm H_2_O (group B). At baseline, there were no significant differences in PaO_2_/FiO_2_-ratio between group A and group B (p = 0.779). Dynamic respiratory system compliance was also not different between groups (p = 0.075). After EIT guided PEEP adjustment both PaO_2_/FiO_2_-ratio and Cdyn increased significantly in both groups (p < 0.001). However, for both these parameters, there were no significant differences in effect size between both groups (p = 0.894 and p = 0.151 respectively). Effects of EIT-guided PEEP adjustment on PaO_2_/FiO_2_-ratio and Cdyn are presented in Figs. 6 and 7.

**Fig. 8:**
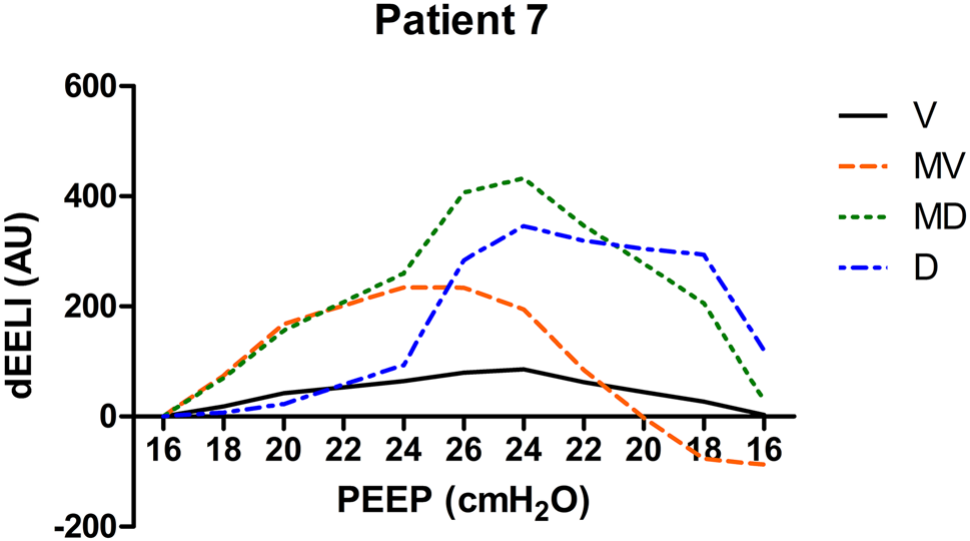
Changes in EELI in four regions of regions of interest during the PEEP trial (*V* ventral, *MV* mid-ventral, *MD* mid-dorsal, *D* dorsal). There is a large drop of EELI in the mid-dorsal and dorsal region when PEEP was decreased below 18 cm H_2_O. PEEP was set at 20 cm H_2_O in this patient (*dEELI* delta-end expiratory lung impedance, *AU* arbitrary units)

**Fig. 9:**
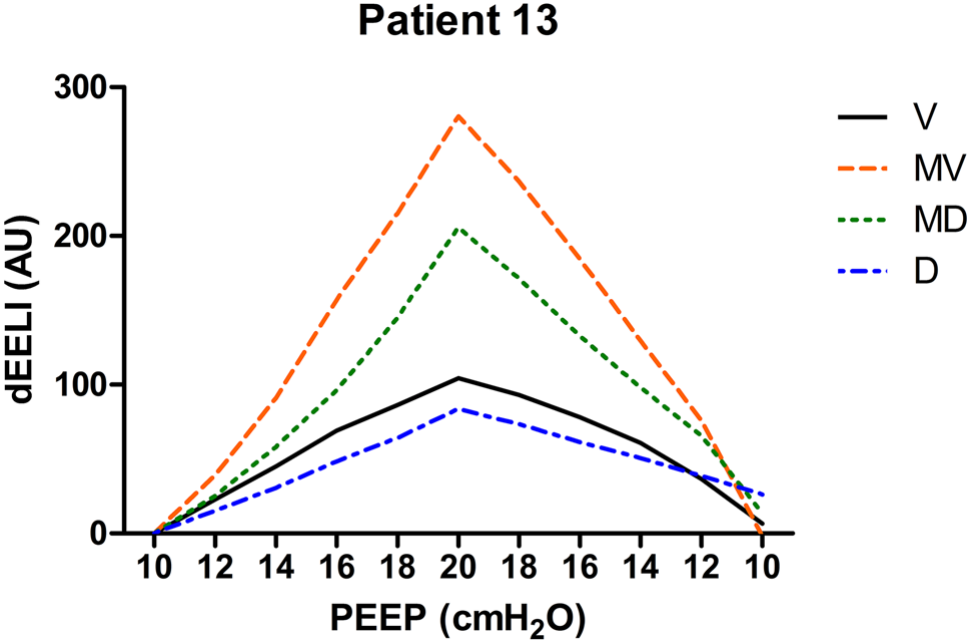
Changes in EELI in four regions of regions of interest during the PEEP trial (*V* ventral, *MV* mid-ventral, *MD* mid-dorsal, *D* dorsal). At the end of the PEEP trial EELI returns to baseline. PEEP was set at 10 cm H_2_O in this patient (*dEELI* delta-end expiratory lung impedance, *AU* arbitrary units)

**Fig. 10:**
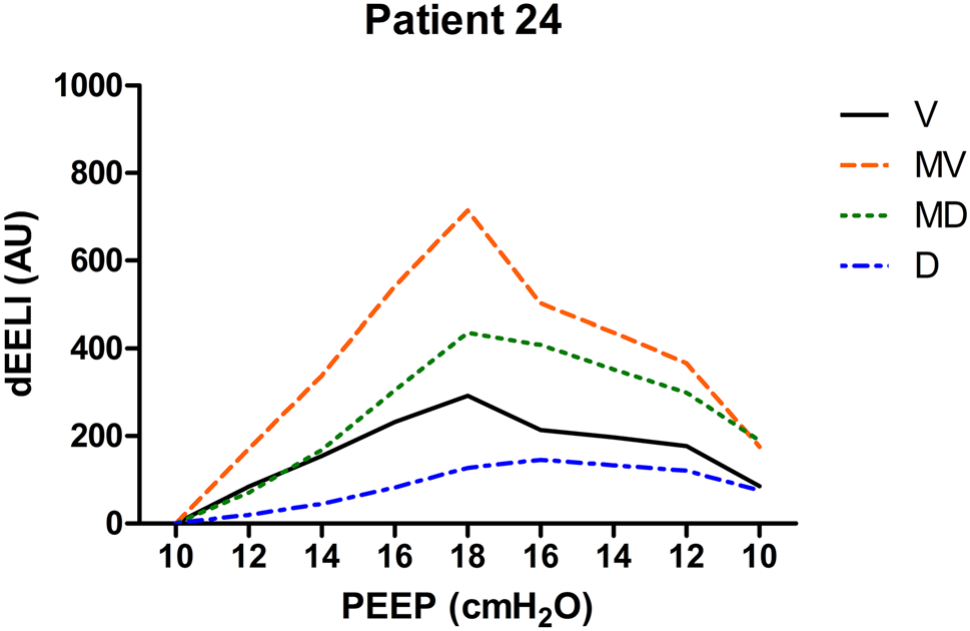
Changes in EELI in four regions of regions of interest during the PEEP trial (*V* ventral, *MV* mid-ventral, *MD* mid-dorsal, *D* dorsal). At the end of the PEEP trial EELI is higher compared to baseline. PEEP was set at 10 cm H_2_O in this patient (*dEELI* delta-end expiratory lung impedance, *AU* arbitrary units)

Individual EELI tracings from three patients during the PEEP trial in four regions of interest are presented in Figs. 8, 9 and 10.

## Discussion

This is the first study reporting on the results of routine clinical use of EIT to guide PEEP setting in mechanically ventilated ARDS patients. We found that in approximately two-thirds of the patients, EIT guided PEEP differed from physicians’ set PEEP and from ARDS network suggested PEEP. In approximately one-third of the patients the PEEP difference was even 4 cm H_2_O or more.

To this point there is no definition for optimal PEEP, there is no consensus whether to use high PEEP levels to avoid all lung collapse or if overdistension is a contributive factor to VALI. At this time there are different views on the matter. Lachmann introduced the “open lung concept” applying higher levels of PEEP to keep the lungs open while others focus their ventilator settings on avoiding overdistension secondary to high PEEP levels, or define optimal PEEP as the lowest level of alveolar overdistension and collapse [19–22]. The PEEP level using a more open lung approach with CL less than 3 or 1% are presented as supplementary material.

The present study shows a clear difference in set PEEP comparing the three methods (PEEP according to EIT, ARDS network table and clinician-based PEEP), without proving that the one method is better than the other. From a physiological point of view, an individualized approach which adjusts delta pressure, PEEP and respiratory rate to a patients’ specific lung mechanics seems appropriate. In literature, different responses to PEEP steps are found between different patient groups but also within groups [23]. The PEEP/FiO_2_-table from the ARDS network study is a generalized PEEP approach, not adapted to the patients specific characteristics, which might explain the differences between EIT guided PEEP in our study group versus ARDS network table’s guided PEEP.

Using changes in respiratory system compliance during a PEEP trial seems to be an useful alternative to determine the optimal PEEP as described earlier [7, 24]. The present study shows that in almost 50% of the patients EIT-guided PEEP was equal to the PEEP level which corresponds with the best Cdyn. However, in 18% of the patients the difference in optimal PEEP was 4 cm H_2_O, however never bigger. The advantage of EIT is that it can identify the level of PEEP where derecruitment begins, even if global Cdyn still increases due to some relief of OD, which can also be visualised with EIT.

Changes in EELI have different patterns in different patients as shown in Figs. 8, 9, 10. The increase in EELI was not always preferentially distributed to the non-dependent lung regions as Lowhagen et al. showed before [25]. In some patients EELI is increased after the PEEP trial reflecting alveolar recruitment. Changes in EELI can only be used to estimate changes in end expiratory lung volume since EIT is measured at only one level just above the diaphragm. However, there is a moderate agreement between end expiratory lung volume and the volume calculated from changes in EELI [26].

Global measures of oxygenation or respiratory system mechanics are usually applied as reference to adjust mechanical ventilation on an individual basis. These global measures may produce misleading information by ‘averaging’, they do not exclude overdistension, tidal recruitment or collapse in different lung regions [27]. Radiological images are very useful as a diagnostic tool for pulmonary edema, pleural effusion, pneumothorax, atelectasis, etc., but they are limited because only one point in time is obtained. Changes that occur dynamically will be missed. Other methods to determine PEEP like low-flow pressure–volume curves, stress-index, oesophageal pressure, PEEP/FiO_2_-tables do not take into account regional overdistension or collapse, they lack information on ventilation distribution. Only CT-scan, with the exception of EIT, can provide this information but still is one sample in time. An individualized approach while monitoring for regional overdistension, atelectrauma and alveolar collapse is probably superior to an approach not taken into account the individual characteristics of each patient. EIT offers the ability to monitor regional respiratory mechanics and monitor its clinical course as compared to the global measures of respiratory system mechanics that are commonly clinically assessed at the bedside to guide the ventilator settings [27]. Also, EIT can monitor regional compliance. During changes in PEEP, the interaction between dependent and non-dependent regions can be disclosed [23]. The assessment of regional overdistension and atelectasis can be visualized using EIT during changes in PEEP and has been validated by histological examination of lung tissue [28]. An animal study used by Wolf et al. was the first prospective study wherein EIT guided ventilator settings were used [18], followed by other animal and human studies [22, 29].

In the present study we assessed short term clinical effects of PEEP adjustment. Whereas PEEP is an important determinant of oxygenation and compliance in the short term, the effects of inadequate ventilation may only become overt weeks to months after the acute phase of ARDS in the form of VALI. EIT may play an important role in the prevention of VALI by minimizing repetitive alveolar collapse or overdistension. Such long term effects can only be studied in a prospective randomized trial with long-term follow-up.

After PEEP-adjustment according to the EIT findings, we observed a significant direct increase in oxygenation and dynamic compliance. However, this increase was also observed in the group where the PEEP difference was less than 4 cm H_2_O (group A). We hypothesize that the incremental and decremental PEEP trial that was performed during the EIT study had similar effects as a lung recruitment manoeuvre. Due to the study method we were not able to extend our comparative follow up afterwards since PEEP remained at the level set according to EIT-advise. This eliminated the possibility to compare ventilation and oxygenation between patients who are treated using EIT guidance and a control group where an alternative method of PEEP setting was applied.

EIT analyses depend on the experience of the operator and thus are vulnerable in terms of bias. Moreover, not visualizing ventilation distribution in the whole lung is a drawback of EIT measurements. The impedance changes are measured in a lens-shaped slice of the thorax with an increase in thickness of approximately 12 cm in the central region of the body [30]. The position of the belt effects the EIT measurements. In mechanically ventilated patients, the PEEP level with the best regional compliance is different for the dependent and nondependent lung regions as well as for the caudal and cranial lung levels. When decreasing PEEP while EIT is measured at the caudal lung level, the diaphragm might enter the measurement field causing artefacts and unreliable results [31]. Therefore, new analysis tools and algorithms that extract clinically relevant information and reduce the clinicians workload are necessary to facilitate the clinical application and implementation in daily care of EIT. In this study we started with an incremental PEEP trial at low PEEP, hereby artefacts caused by diaphragm displacement could be detected and the position of the belt corrected.

To study the effect of EIT guidance in PEEP optimization, prospective randomized trials comparing different alternative approaches such as the ARDS network table to determine PEEP settings with EIT in ARDS patients are warranted. Especially since this study showed a very limited agreement between PEEP levels advised by the ARDS network table and by EIT.

## Conclusions

This is a unique series of patients with ARDS in which EIT is used to guide ventilatory settings in practice. Our single center experience shows EIT can be used in the clinical setting. As regional overdistension and alveolar collapse can be visualized using EIT, it is a promising tool which has a large potential for becoming the golden standard as a bedside patient-tailored ventilatory setting tool. A randomized controlled trial with long term outcome effects is required to see if EIT guided PEEP setting actually improves clinical outcome.

## Electronic supplementary material

Below is the link to the electronic supplementary material.

Supplementary material 1 (XLSX 12 KB)

## Abbreviations

ABG: Arterial blood gas
ANOVA: Analysis of variance
APACHE: Acute physiology and chronic health evaluation
ARDS: Acute respiratory distress syndrome
BMI: Body mass index
Cdyn: Dynamic respiratory system compliance
EIT: Electrical impedance tomography
ICU: Intensive care unit
PEEP: Positive end expiratory pressure
SD: Standard deviation
VALI: Ventilator associated lung injury
